# Stretching human mesenchymal stromal cells on stiffness-customized collagen type I generates a smooth muscle marker profile without growth factor addition

**DOI:** 10.1038/srep35840

**Published:** 2016-10-24

**Authors:** Miriam Rothdiener, Miriam Hegemann, Tatiana Uynuk-Ool, Brandan Walters, Piruntha Papugy, Phong Nguyen, Valentin Claus, Tanja Seeger, Ulrich Stoeckle, Karen A. Boehme, Wilhelm K. Aicher, Jan P. Stegemann, Melanie L. Hart, Bodo Kurz, Gerd Klein, Bernd Rolauffs

**Affiliations:** 1 Siegfried Weller Institute for Trauma Research, BG Trauma Clinic Tuebingen, University of Tuebingen, Germany; 2 Department of Urology, University of Tuebingen, Germany; 3 Department of Biomedical Engineering, University of Michigan, Ann Arbor, MI, USA; 4 Center for Medical Research, Medical University Clinic II, University of Tuebingen, Germany; 5 Clinic for Trauma and Restorative Surgery, BG Trauma Clinic Tuebingen, University of Tuebingen, Germany; 6 Department of Orthopaedic Surgery, University of Tuebingen, Germany; 7 Department of Orthopedics and Trauma Surgery, Albert-Ludwigs-University, Freiburg, Germany; 8 Department of Anatomy, Christian-Albrechts-University, Kiel, Germany

## Abstract

Using matrix elasticity and cyclic stretch have been investigated for inducing mesenchymal stromal cell (MSC) differentiation towards the smooth muscle cell (SMC) lineage but not in combination. We hypothesized that combining lineage-specific stiffness with cyclic stretch would result in a significantly increased expression of SMC markers, compared to non-stretched controls. First, we generated dense collagen type I sheets by mechanically compressing collagen hydrogels. Atomic force microscopy revealed a nanoscale stiffness range known to support myogenic differentiation. Further characterization revealed viscoelasticity and stable biomechanical properties under cyclic stretch with >99% viable adherent human MSC. MSCs on collagen sheets demonstrated a significantly increased mRNA but not protein expression of SMC markers, compared to on culture flasks. However, cyclic stretch of MSCs on collagen sheets significantly increased both mRNA and protein expression of α-smooth muscle actin, transgelin, and calponin versus plastic and non-stretched sheets. Thus, lineage-specific stiffness and cyclic stretch can be applied together for inducing MSC differentiation towards SMCs without the addition of recombinant growth factors or other soluble factors. This represents a novel stimulation method for modulating the phenotype of MSCs towards SMCs that could easily be incorporated into currently available methodologies to obtain a more targeted control of MSC phenotype.

Worldwide, mesenchymal stromal cells (MSCs) are the center of intensive efforts to develop cell-based therapies for a diverse range of diseases^1^. Bone marrow MSCs promise a variety of clinical applications because they can differentiate *in vitro* towards cell types of mesodermal origin including osteogenic, chondrogenic, adipogenic and myogenic lineages^2^,^3^, and also into neurons^4^, vascular endothelial cells^5^, and cardiomyocytes^6^. A key aspect to making MSC-based therapies a clinical reality will be to better understand and manipulate the interactions between the MSCs and their local *in vivo* and *in vitro* microenvironment in order to control MSC fate^7^,^8^,^9^,^10^.

A growing number of studies employ biophysical cues to improve currently available methods for investigating and steering MSC differentiation. Biomaterials with defined stiffnesses are a promising tool in this application^11^,^12^ because MSC lineage commitment can be enhanced by specific matrix elasticity. For example, lineage-specific microenvironment elasticities of 0.1–1 kPa, 8–17 kPa, and 25–40 kPa promote high expression levels of neurogenic, myogenic, and osteogenic markers, respectively^13^. Other attractive biophysical tools are biomechanical forces, to which undifferentiated human MSCs are highly sensitive^14^. Previous studies have applied cyclic biomechanical stretch to generate myogenically differentiating MSCs^15^,^16^,^17^, based on the observation that an elongated MSC morphology is associated with increased expression of smooth muscle cell (SMC) markers^18^. However, cyclic stretch has not been used together with lineage-specific stiffness for inducing myogenic differentiation of MSCs.

In the present study, we hypothesized that combining lineage-specific stiffness with cyclic stretch may result in a significantly enhanced SMC phenotype, without the need for exogenous growth factors. Accordingly, we investigated the effects of lineage-specific stiffness with and without cyclic stretch on the differentiation of MSCs towards SMCs. A previous study has shown that a stiffness range of 8 to 17 kPa is supportive of myogenic differentiation^13^. We therefore generated physiologically relevant substrates with stiffness in this range by mechanically compressing collagen type I hydrogels into dense sheets, termed compressed collagen^19^. This type of material has been used previously for generating artificial corneas^20^,^21^, skin^22^, bone^23^, and bladder^24^. The substrate collagen type I was chosen because this biomaterial has already been used for applying cyclic stretch with the goal of inducing myogenic differentiation in MSCs^15^,^17^,^25^,^26^,^27^. We characterized the compressed collagen in terms of the survival of adhering MSCs, nano- and microscale biomechanical properties, loss of stiffness during repetitive tensile loading, and surface topography. We then applied defined cyclic stretch to the compressed collagen sheets and the adhering human bone marrow derived MSCs and assessed the expression of specific markers associated with the SMC phenotype. The ability to reliably differentiate MSCs into SMCs would be an important tool in understanding phenotype shifts in these cells, and would advance the fields of vascular biology and tissue engineering.

## Results

### Characterization of compressed collagen type I sheets

**Figure 1:**
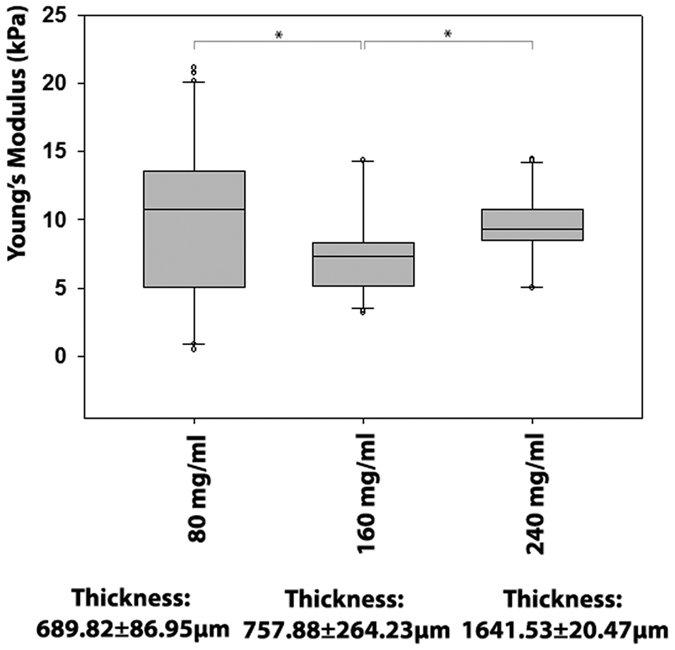
Nanoscale biomechanical properties assessed by atomic force microscopy (AFM). The stiffness of compressed collagen type I sheets with 160 mg/ml collagen concentration was significantly lower than the stiffness of sheets with 80 mg/ml and 240 mg/ml collagen concentrations. However, all collagen type I sheets were in or near the nanoscale stiffen ss range of 8 to 17 kPa desired for myogenic differentiation. *Indicate significant differences. Data are presented as boxplot; the line within the box marks the median, the boundary of the box indicates the 25^th^ and 75^th^ percentile. The error bars above and below the box indicate the 90^th^ and 10^th^ percentile, and the dots above and below the error bars represent outlying points. Number of individually performed experiments: n = 3 sheets for each group, measured in triplicates at three locations for each sheet.

Testing of the nanoscale mechanical properties of compressed collagen sheets (n = 3 sheets for each collagen concentration) was performed with atomic force microscopy (AFM; n = 3 locations per sheet, measured in triplicate). AFM revealed that all collagen type I sheets were in a relatively narrow stiffness range from approximately 7–11 kPa (Fig. 1). However, there were differences in the nanoscale stiffness between the sheets, and the 160 mg/ml collagen type I sheet stiffness (Young’s Modulus: 7.2 ± 0.6 kPa) was significantly lower than the 80 mg/ml (10.2 ± 0.8 kPa, p < 0.001) and 240 mg/ml sheets (9.7 ± 0.5 kPa, p < 0.001).

**Figure 2:**
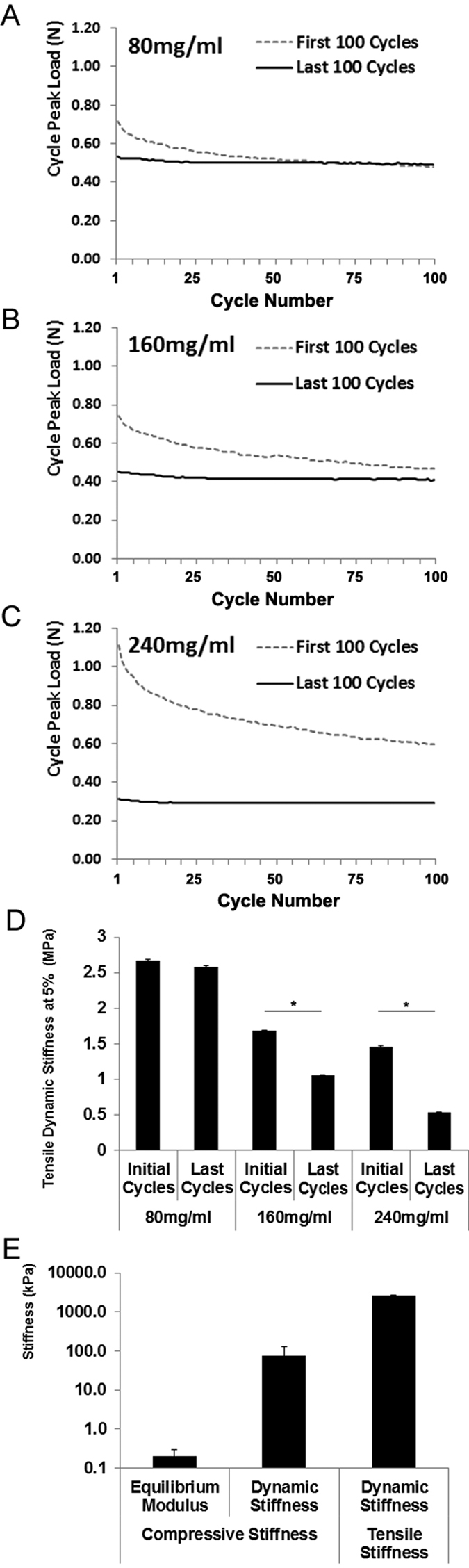
Fatigue behavior and microscale tensile and compressive biomechanical properties of compressed collagen sheets. (**A**) To characterize fatigue behavior, the cycle peak load of the first and the last 100 cycles of 1 hour stretch (5%, f = 1 Hz) was recorded for the 80 mg/ml collagen type I sheets. The data indicate a decrease in the recorded peak load that occurred approximately during the first 50 cycles. No further decrease occurred when assessing the remaining cycles of the first 100 cycles analyzed, compared to the last 100 cycles analyzed. This suggested a certain degree of material fatigue during the first 50 cycles but not during later cycles of 1 hour stretch. (**B**,**C**) The corresponding cycle peak load for the 160 mg/ml and the 240 mg/ml collagen type I sheets indicated that 160 mg/ml sheets (**B**) and more so 240 mg/ml sheets (**C**) exhibited a decrease in peak load throughout all first 100 cycles of stretch. The peak load of the last 100 cycles of stretch was lower than any load recorded during the first 100 cycles, indicating that both the 160 mg/ml and the 240 mg/ml collagen sheets exhibited a much higher degree of material fatigue over a longer period of time than the 80 mg/ml sheets. (**D**) When assessing the tensile stiffness during the first and last 100 cycles of 1 hour of stretch (5%, 1 Hz), compressed collagen type I sheets with 80 mg/ml had retained 96.7% of their tensile stiffness, while 160 mg/ml sheets had retained 62.4% and 240 mg/ml sheets had retained 36.0%. (**E**) Characterization of the microscale compressive properties of collagen type I sheets (80 mg/ml) revealed that the equilibrium modulus was approximately two orders of magnitude lower than the dynamic stiffness. The tensile dynamic stiffness was higher than both the compressive equilibrium modulus and the compressive dynamic stiffness. Data in (**A**–**C**) is presented as mean, data in (**D**,**E**) is presented as mean ± SEM. Number of individually performed experiments: n = 3 sheets. * indicate significant differences.

**Figure 3:**
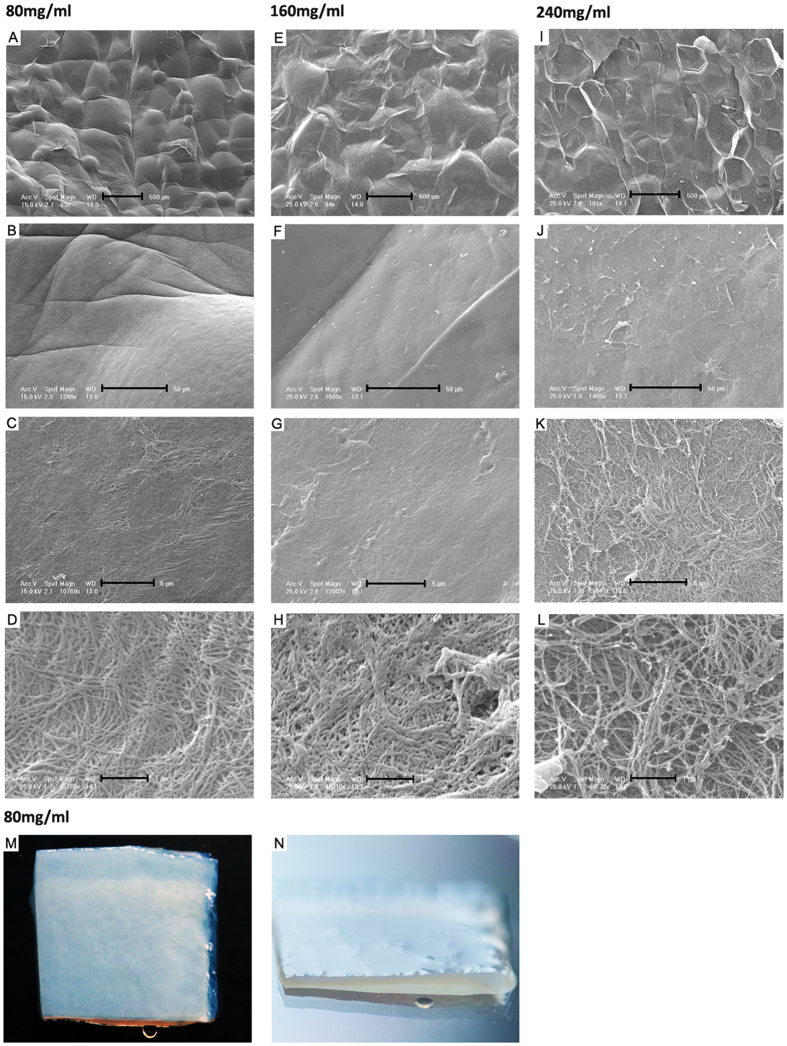
Surface topography of collagen type I sheets assessed via scanning electron microscopy (SEM) and gross appearance via macro photography. Compressed collagen type I sheet surfaces of a concentration of 80 mg/ml exhibited at relatively low magnifications of 83x (**A**) and 1346x (**B**) a non-homogeneously structured surface with a leaf-like topography. At magnifications of 10769x (**C**) and 48788x (**D**) collagen fibers were visible. Compressed collagen type I sheet surfaces of a concentration of 160 and 240 mg/ml exhibited comparable characteristics at low magnifications (160 mg/ml: (**E**,**F**); 240 mg/ml: (**I**,**J**) and high magnifications (160 mg/ml: (**G**,**H**); 240 mg/ml: (**K**,**L**). The gross appearance of compressed collagen type I sheets of a concentration of 80 mg/ml is depicted in a top-down view in (**M**) and in a side view in (**N**). Scale bars, 500 μm (**A**,**E**,**I**), 50 μm (**B**,**F**,**J**), 5 μm (**C**,**G**,**K**), and 1 μm (**D**,**H**,**L**), sample size for (**M**,**N**): 1 cm × 1 cm.

In contrast to compressive stiffness determined with AFM, the tensile stiffness was determined under cyclic tension. To compare the tensile properties of the compressed collagen sheets under repetitive loading, the tensile stiffness was calculated analyzing the first and last 100 cycles of 1 hour sinusoidal stretch at 5% and 1 Hz for sheets with collagen concentrations of 80, 160, and 240 mg/ml. Sheets with 80 mg/ml retained 96.7% of their tensile stiffness. The difference in the tensile stiffness prior and after 1 hour stretch calculated from the first and last 100 stretch cycles was not significant (initial stiffness: 2.67 ± 0.01 MPa, after 100 cycles: 2.58 ± 0.01 MPa, Fig. 2A). Collagen type I sheets with 160 mg/ml retained 62.4% and those with 240 mg/ml retained 36.0% of their tensile stiffness, and the difference in the tensile stiffness prior and after 1 hour stretch was significant for both 160 and 240 mg/ml concentrations (p < 0.001). For assessing the fatigue behavior in more detail, we also analyzed the cycle peak load of the first and last 100 cycles of 1 hour sinusoidal stretch at 5% and 1 Hz. 80 mg/ml collagen type I sheets exhibited a certain degree of material fatigue during the first 50 cycles but not during later cycles of 1 hour stretch (Fig. 2A). Both the 160 mg/ml (Fig. 2B) and the 240 mg/ml collagen sheets (Fig. 2B) exhibited a much higher degree of material fatigue over a longer period of time, compared to the 80 mg/ml sheets. Because of their higher property retention, the 80 mg/ml compressed collagen sheets were chosen for use in the mechanical strain studies, and were characterized further. Thus, their macroscale compressive properties were determined (equilibrium modulus: 0.21 ± 0.09 kPa, dynamic stiffness: 76.8 ± 51.9 kPa). A small phase lag between strain and stress was observed under dynamic compression and also during the dynamic tensile tests, confirming that the compressed collagen sheets have viscoelastic properties. Scanning electron microscopy was used to characterize the surface topography of the compressed collagen sheets (Fig. 3). For all collagen concentrations used (80, 160, and 240 mg/ml), the sheet surfaces displayed a featured surface with a hill- and valley-like topography at lower magnifications (approximately 80–100x, Fig. 3A,E,I, and 1300–1500x, Fig. 3B,F,J). At higher magnifications (approximately 10000x-16000x, Fig. 3C,G,K) the topography was less evident. At very high magnifications (approximately 48000x, Fig. 3D,H,L) the network of collagen fibers and nanoscale topology that made up the compressed collagen sheets was visible. The photographed gross appearance of the 80 mg/ml compressed collagen type I sheets is shown in Fig. 3M,N. The gross appearance of 160 and 240 mg/ml compressed collagen type I sheets was very similar to that of the 80 mg/ml sheets (data not shown).

### MSC characterization, seeding and culture

The MSCs used in this study fulfilled the criteria for defining multipotent MSCs according to the guidelines proposed by the Mesenchymal and Tissue Stem Cell Committee of the International Society for Cellular Therapy (ISCT)^28^. They were plastic-adherent, expressed CD90, CD105, CD73, but not CD14, CD11b, CD45 and CD34. As we have previously demonstrated, MSCs could be differentiated *in vitro* into the adipogenic, osteogenic and chondrogenic lineages using standard protocols and biochemically defined media^29^,^30^.

**Figure 4:**
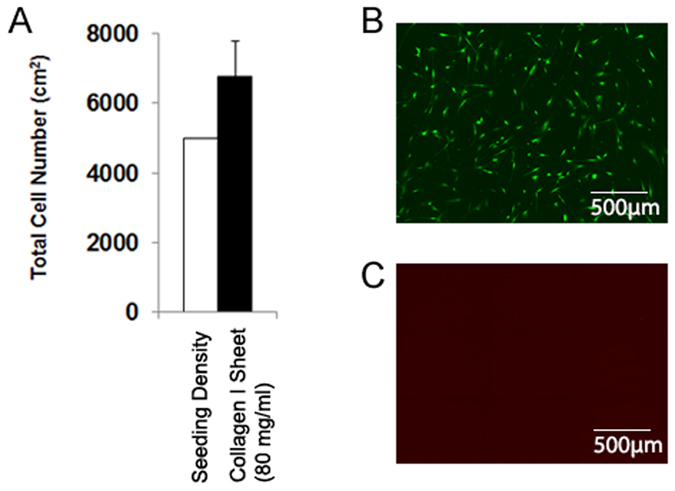
Cell numbers and vitality of adherent MSCs. (**A**) The number of MSCs that adhered to the compressed collagen type I sheets at day 4 was not significantly different from the day 0 seeding density. (**B**) 99.9% of adherent MSCs were vital, which is illustrated by the representative fluorescence-microscopy images of calcein (green, live MSCs) and (**C**) propidium iodide (red, dead MSCs) stained MSCs. Number of patients and individually performed experiments: n = 5.

The numbers of MSCs that adhered to the 80 mg/ml collagen type I sheets (6770 ± 1021) were not significantly different from the MSC seeding density (Fig. 4A). Additionally, viability of MSCs on compressed collagen type I sheets was very high. At day 4, viability was >99% in all samples (Fig. 4B).

### Characterization of MSC phenotype on compressed collagen sheets

**Figure 5:**
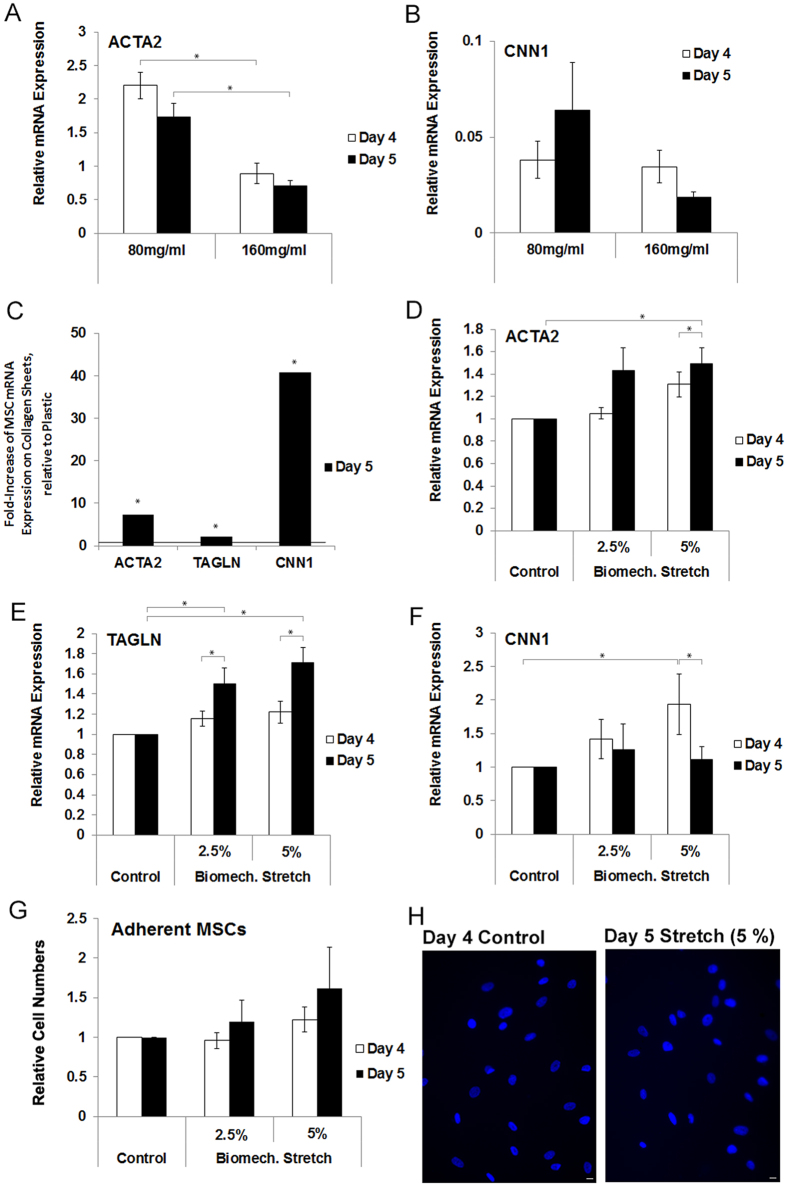
Gene expression profiles of stimulated MSCs on compressed collagen type I sheets, MSC numbers, and representative images of DAPI stained nuclei for assessing cell numbers of non-stretched control and 5% stretched MSCs. (**A**) When assessing the effects of two different collagen type I concentrations, a lower collagen concentration was associated with a significantly higher mRNA expression of ACTA2. Without stretch, time (day 4 vs. 5) was associated with a significantly lower gene expression of ACTA2 but the effects of time were less than the effects of collagen concentrations. (**B**) A trend towards a higher CNN1 mRNA expression was observed on 80 mg/ml collagen type I sheets but this change did not reach significance. (**C**) Compared to MSCs on plastic, MSCs on non-stretched collagen sheets exhibited a significant 7.4 fold increase of ACTA2, 2.2 fold increase of TAGLN, and a significant 40.8 fold increase of CNN1 mRNA expression (p < 0.01). (**D**–**F**) MSCs that were biomechanically stimulated via cyclic stretch (2.5 and 5% amplitude and 1 Hz frequency) demonstrated a significantly higher gene expression of ACTA2 (**D**) on day 5 under 5% stretch, TAGLN (**E**) on day 5 under both 2.5% and 5% stretch, and CNN1 (**F**) on day 4 under 5% stretch. Thus, biomechanical stretch of 5% induced the gene expression of the myogenic marker genes ACTA2, TAGLN, and CNN1. With 5% stretch, time (day 4 vs. 5) was associated with a significantly increased ACTA2 and TAGLN and significantly decreased CNN1 mRNA expression. (**G**) Cyclic stretch did not significantly change the numbers of MSCs. (**H**) Representative images of DAPI stained MSC nuclei, used for assessing the cell numbers of non-stretched control MSCs and 5% stretched MSCs. *Indicate significant differences. Data in (**A**,**B**,**D**–**G**) is presented as mean ± SEM, in C as fold-change. Number of individually performed experiments/samples: n = 3 for (**A**,**B**), n = 6 for MSC cultures on plastic in (**C**), n = 12 for (**D**–**G**). Scale bar (**H**): 10 μm.

We chose sheets with collagen concentrations of 80 and 160 mg/ml to compare the effect of different substrate concentrations on the relative gene expression using qRT-PCR. ACTA2 expression was significantly higher (p < 0.001) on 80 mg/ml compressed collagen sheets (2.20 ± 0.21 vs 0.89 ± 0.16, day 4, Fig. 5A). Over time (from day 4 to 5), ACTA2 expression decreased significantly (p < 0.001) on sheets with both concentrations (80 mg/ml: 2.20 ± 0.21 vs 1.73 ± 0.22, 160 mg/ml: 0.89 ± 0.16 vs 0.70 ± 0.08). However, the difference between both sheet types on day 5 remained statistically significant (p < 0.001). The relative gene expression of CNN1 was not significantly different between the two sheets or between days 4 and 5 (Fig. 5B). Next, MSC mRNA expression was compared on day 5 between cell culture flasks (n = 6 cultures) and 80 mg/ml collagen type I sheets. MSCs adhering to the collagen sheets exhibited a significant 7.4 fold increase of ACTA2, a significant 2.2 fold increase of TAGLN, and a significant 40.8 fold increase of CNN1 mRNA expression, compared to MSCs on plastic (p < 0.01, Fig. 5C). The relatively high CNN1 increase of mRNA expression was explained by the low CNN1 mRNA expression that MSCs exhibited on plastic.

MSCs (n = 12 bone marrow samples of n = 7 donors, 61 to 80 years of age, average age of 68 years) were cyclically stretched via 2.5 and 5% stretch (1 hour/day on days 4 and 5 at a frequency of 1 Hz) using only the 80 mg/ml collagen type I sheets, and qRT-PCR was performed on stretched and control MSCs. The ACTA2 mRNA expression was significantly increased on day 5 with 5% stretch (1.49 ± 0.14-fold) compared to non-stretched controls (Fig. 5D, p <0.05). The TAGLN mRNA expression was significantly increased on day 5 with 2.5% and 5% stretch (1.22 ± 0.11, 1.71 ± 0.14) compared to non-stretched controls (Fig. 5E, p < 0.05). The CNN1 mRNA expression was significantly increased on day 4 with 5% stretch (1.93 ± 0.45) compared to non-stretched controls (Fig. 5F, p < 0.05). These values were normalized to day 4 and day 5 controls, and both controls were set to a value of 1 because a) their absolute values were comparable, b) their variation was low, and c) there was no significant difference between the control values on days 4 and 5.

When using the reported data for calculating their values relative to SMCs as positive control, the ACTA2 mRNA expression of control MSCs on non-stretched collagen sheets reached 30.3% of SMCs. This result was comparable to the reported 20% ACTA2 expression of control MSCs relative to SMCs^25^. However, the ACTA2 mRNA expression of 5% stretched MSCs on day 5 reached 41% of the human bladder-derived smooth muscle cells, indicating that the ACTA2 expression of MSCs reported in the literature can potentially be doubled by using collagen type I sheets together with cyclic stretch.

We determined the number of stretched or control MSCs per normalized area at the same time points that were analyzed for gene and protein expression under biomechanical stretch (Fig. 5G). However, there were no significant changes in cell numbers per normalized area. Representative images are given in Fig. 5H.

**Figure 6:**
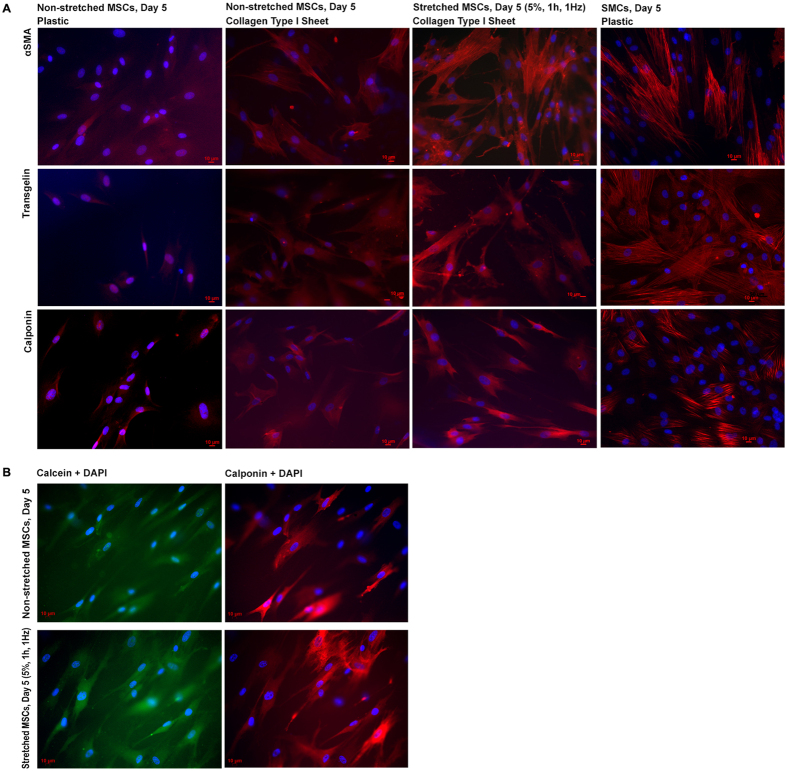
Representative examples of protein expression profiles of MSCs adhering to plastic, non-stretched and stretched collagen type I sheets, and of SMCs adhering to plastic. (**A**) MSCs adhering to plastic exhibited the lowest amounts of αSMA, transgelin and calponin protein, compared to MSCs on collagen type I sheets and SMCs on plastic. MSCs on cyclically stretched collagen type I sheets exhibited the highest amounts of αSMA, transgelin and calponin protein, compared to non-stretched MSCs on collagen type I sheets, and also compared to MSCs on plastic. SMCs on plastic used as positive control exhibited proteins levels that were relatively similar to the stretched MSCs. (**B**) DAPI, calcein, and calponin stained MSCs were visualized in green, blue, and red channels. The recorded images illustrate that most, but not all DAPI and calcein positive MSCs also stained positive for calponin. Thus, calponin-positive but also calponin-negative MSCs were present on non-stretched and on stretched collagen type I sheets. Number of individually performed experiments (**A**): n = 12 (non-stretched and stretched collagen type I sheets), n = 3 (MSCs on plastic, SMCs).

**Figure 7:**
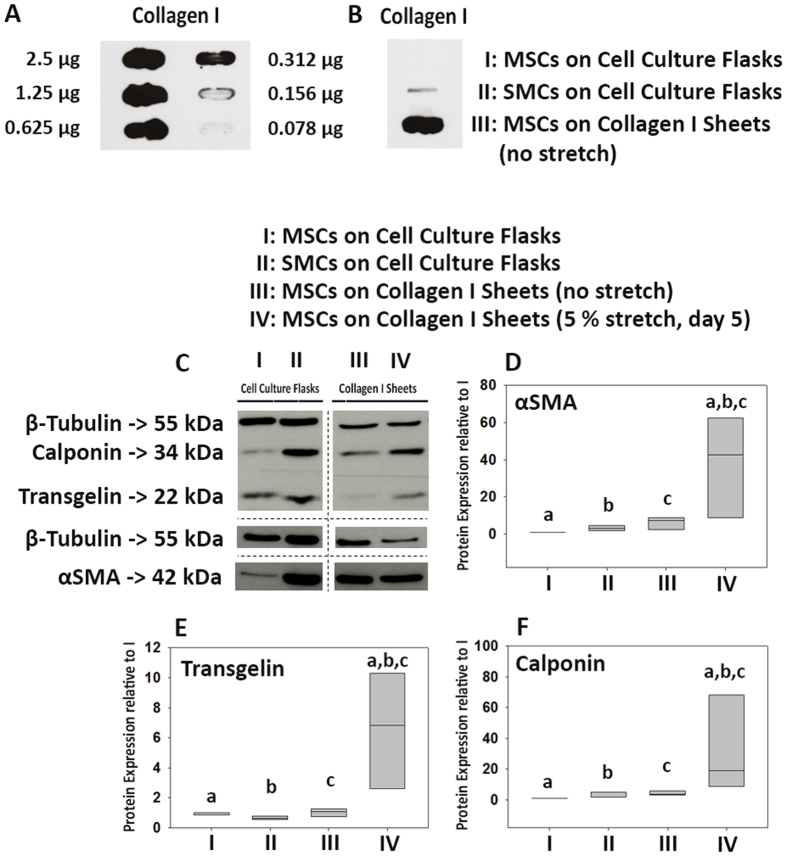
Slot blot of collagen type I for MSCs on collagen type I sheets and Western blot of αSMA, transgelin, and calponin protein expression for MSCs and SMCs on cell culture flasks and MSCs on stretched and non-stretched collagen type I sheets. (**A**) To determine the sensitivity of the slot blot, serial dilutions of collagen type I were investigated. (**B**) The slot blot indicated the presence of collagen protein in samples from cells on collagen type I sheets but not on plastic. This indicated that collagen protein was scratched off the sheets during MSC removal and would bias protein amount determination by immunoblotting. Thus, the additional collagen protein resulted in apparently lower non-collagenous protein amounts in samples from collagen sheets. (**C**) This resulted in a lower β-tubulin signal from MSCs derived from collagen sheets, despite Bradford protein assay-controlled protein loading. We accounted for this bias by normalizing the αSMA, transgelin, and calponin protein amounts to β-tubulin. Then, we calculated the fold-change of these proteins relative to MSCs on cell culture flasks. (**D**–**F**) The highest amounts of αSMA, transgelin, and calponin protein were present in MSCs cultured on collagen type I sheets subjected to cyclic stretch. This was confirmed by statistical analyses, which revealed that the protein amounts of these 3 markers were significantly higher in MSCs from cyclically stretched collagen type I sheets (p < 0.001) than from all other groups. Number of individually performed experiments: n = 4. The Western blots depicted in (**C**) have been run under the same experimental conditions. The depicted lanes for β-tubulin, calponin, and transgelin were cropped from a single Western blot, and the lanes depicted for αSMA and β-tubulin were cropped from another Western blot before (αSMA) and after (β-tubulin) stripping. Cropping lines are indicated by dotted lines. Both original Western blots are given in full length in the supplementary information. Significant differences between groups are indicated by small letters, e. g. in (**D**) “a” indicates that αSMA protein was significant lower in samples from MSCs that were cultured on plastic (**D**,**I**) than on cyclically stretched collagen type I sheets (**D**, VI).

Next, we determined the protein levels using immunofluorescence microscopy. MSCs that adhered to plastic exhibited visibly less αSMA, transgelin and calponin protein, compared to MSCs on non-stretched collagen type I sheets (Fig. 6A). However, MSCs on 5% stretched collagen type I sheets showed stronger signals for αSMA, transgelin and calponin protein, compared to non-stretched MSCs on collagen type I sheets, and also compared to MSCs on plastic. Thus, using collagen type I sheets for MSC culture led to a slight increase of αSMA, transgelin and calponin protein. Cyclic stretch of MSCs on collagen type I sheets visibly increased the amount of αSMA, transgelin and calponin protein, which, based on immunofluorescence, reached relatively similar levels of SMCs used as positive control. Interestingly, the majority but not all SMCs stained positive for the contractile markers αSMA and CNN1. This is in line with the fact that SMCs can undergo phenotypic changes from a contractile phenotype to a proliferative ‘synthetic’ phenotype^31^. Motivated by this observation, we investigated whether MSCs displayed a comparable behavior. Virtually all MSCs on non-stretched and stretched collagen type I sheets stained positive for αSMA and transgelin but not all MSCs stained positive for calponin (Fig. 6B). Thus, we calculated the percentage of calponin-positive MSCs and found that 80.2 ± 6.8% MSCs on non-stretched collagen type I sheets and 83.2 ± 3.0% MSCs on stretched collagen type I sheets were calponin-negative. This is in line with what is found for SMCs *in vitro* and *in vivo*, which can exist as a continuum of phenotypes^32^,^33^. Next, we assessed protein levels by Western blotting. We observed that the tubulin amount was lower in samples derived from collagen type I sheets, despite determining correct loading via Bradford protein assay. We suspected that collagen protein scratched from the collagen sheets during MSC removal had biased the determination of protein amounts in extracts of cells grown on and scratched from collagen scaffolds but not from cell culture flasks. This was confirmed by slot blot analysis (Fig. 7A,B). The additional collagen protein resulted in apparently lower non-collagenous protein amounts in samples from collagen sheets, which was illustrated by a lower β-tubulin signal from MSCs derived from collagen sheets despite Bradford protein assay-controlled protein loading. We accounted for this bias by normalizing the αSMA, transgelin, and calponin protein amounts to β-tubulin. Subsequent quantification, and statistical analyses demonstrated that the protein amounts of αSMA, transgelin and calponin were not significantly different between MSCs on cell culture flasks vs. collagen type I sheets and between MSCs vs. SMCs on cell culture flasks. However, the highest protein amounts of αSMA, transgelin and calponin were present in MSCs that were cultured on 5% stretched collagen type I sheets, and these amounts were significantly higher than in any other group (p < 0.001, Fig. 7C–F). Thus, cyclic stretch did significantly increase the protein expression of SMC markers in MSCs that were cultured on collagen type I sheets.

## Discussion

The present study combined lineage-specific surface stiffness with cyclic stretch to induce differentiation of human MSCs towards a SMC-like phenotype without adding recombinant growth factors. We demonstrated that the mechanical compression of collagen type I hydrogels by weights generated dense sheets of compressed collagen with a surface nanoscale stiffness known to support myogenic differentiation. When using these sheets, the mRNA expression of the smooth muscle genes αSMA, transgelin, and calponin was significantly increased, compared to plastic. Using the collagen sheets in combination with cyclic stretch significantly increased the mRNA expression of these markers even further and also resulted in a significant increase in protein levels, suggesting an enhanced SMC phenotype. Thus, the present study established a novel, growth factor-free MSC stimulation method by combining matrix stiffness and biomechanical loading.

Previous studies applied cyclic stretch to MSCs that were seeded on silicone coated with collagen type I^15^,^17^,^25^,^26^,^27^, and on collagen type I hydrogels^27^. Consequently, we chose type I collagen for our study aim. However, collagen hydrogels can be very soft. For example, we calculated relatively low Young’s Moduli of 160 Pa and 390 Pa for hydrogels with collagen concentrations of 3.75 mg/ml^27^ and 4 mg/ml^34^. In contrast, much higher moduli in the range of 2300 kPa can be reached with generating compressed collagen sheets through plastic compression of collagen type I hydrogels^35^, and the mechanical properties of compressed collagen can be engineered to match those found in tissues *in vivo*^36^. Thus, we reasoned that a higher collagen concentration found in compressed collagen may be beneficial for achieving higher stiffness, and we mechanically compressed type I collagen hydrogels for generating dense compressed collagen sheets. AFM measurements demonstrated a nanoscale surface stiffness in the range to support myogenic MSC lineage specification that was comparable across all final collagen concentrations. Having achieved the desired nanoscale stiffness, collagen type I concentrations lower than 80 mg/ml were not generated at this point. Prior to using the compressed collagen sheets for applying cyclic stretch to MSCs, we characterized the sheets in detail. Human MSCs retained a high viability while they adhered to the compressed collagen sheets. Their quantities that were always above the amount seeded, indicating that the compressed collagen sheets were suitable for MSC culture. Next, we investigated the effects of two different collagen type I concentrations on relative gene expression. We chose ACTA2 as marker gene because smooth muscle actin has been frequently used to compare MSC expression to SMCs^25^ under a variety of conditions. We observed a significantly higher expression level of ACTA2 in MSCs on 80 mg/ml collagen type I sheets, compared to 160 mg/ml. This supported the choice of a final type I collagen concentration of 80 mg/ml for subsequent experiments. Next, we characterized the loss of tensile properties during the time period needed for one stimulation cycle. The 80 mg/ml compressed collagen sheets retained 96.7% of their initial tensile properties, whereas higher collagen concentrations were associated with a significantly lower retained percentage of the initial tensile properties. Next, we compared the peak load of 100 loading cycles before and after 1 hour of stretch. The 80 mg/ml collagen type I sheets exhibited a certain degree of material fatigue during the initial stretch cycles whereas both the 160 mg/ml and the 240 mg/ml collagen type I sheets exhibited a much higher degree of material fatigue over a longer period of time. Collagen fibers can display a complex response to uniaxial tension including strain softening, strain hardening, time-dependent recoverable residual strain, dehydration-induced embrittlement, and susceptibility to cyclic fatigue that are dictated by global characteristic dimensions as well as internal structure^37^. Thus, the here reported data may be valuable for generating compressed collagen sheets that are specifically designed for improving tensile performance. Based on these data, we used only 80 mg/ml compressed collagen sheets for all subsequent biomechanical studies, as the loss of tensile properties of 160 mg/ml and 240 mg/ml did not allow applying cyclic stretch without changing properties over time. A broader characterization collagen type I sheets included scanning electron microscopy (SEM) of the sheets surface. SEM revealed for all collagen concentrations (80, 160, and 240 mg/ml) a comparable hill- and valley-like topography on the order of hundreds of micrometers at lower magnifications, and networks of nanoscale collagen fibers were visible at higher magnifications. This is relevant because fiber type and diameter can affect MSC attachment, morphology, proliferation, and differentiation^38^,^39^ through specific cell surface receptors, and the presence of collagen fiber layers is consistent with the literature^36^. Following SEM, we applied compressive testing for assessing the mechanical properties of the compressed collagen sheets. These tests revealed a small phase lag between strain and stress under dynamic compression and also during the dynamic tensile tests, confirming viscoelastic properties of the compressed collagen sheets. This finding is relevant for biomechanical stimulation because both cultured SMCs^40^ and also smooth muscle tissues^41^ exhibit viscoelastic behavior, and it is increasingly being recognized that viscoelastic properties of materials can affect cell behavior^42^. Collectively, our data demonstrated that collagen type I hydrogels can be compressed mechanically into dense sheets of compressed collagen with viscoelastic properties, maintained tensile dynamic stiffness under repetitive loading, lineage-specific nanoscale surface stiffness, and properties that are suitable for MSC culture.

Finally, we used the compressed collagen sheets that were characterized in-depth for applying cyclic stretch to adhering MSCs. Cyclic stretch led to a significant increase in the mRNA expression of αSMA, transgelin, and calponin, indicating an enhanced SMC phenotype. According to the classification published in^32^, this marker mRNA expression represents an intermediate smooth muscle marker profile in human MSCs. Previous studies using biomechanical stretch as a single differentiation stimulus generated an early (αSMA), intermediate (calponin), and late (desmin) marker expression in rat MSCs^15^,^16^,^17^, and an early (αSMA)^43^ and early intermediate marker expression (αSMA and transgelin) in human MSCs^25^. The results of the present study were comparable to those published. However, the stretch used in^15^,^16^,^17^ was applied for a time of 5 to 7 days and generated a more mature profile in rat MSCs than the profile generated in the present study in human MSCs by only 2 × 60 minutes stretch, distributed over 2 days. This indicates that the time, over which a certain stretch regime is applied, is likely a relevant factor for marker profile expression. Our data revealed differential effects of time on the expressed markers: ACTA2 and TAGLN expression was significantly increased on day 5, whereas CNN1 expression was significantly, but transiently increased on day 4. This is in accordance with another report, which recently demonstrated that myogenic marker expression and electrophysiological characteristics can be induced in human MSCs; however, they are not maintained over time and decrease after a certain time period^44^. Moreover, the time during which cyclic stretch is applied appears to be a relevant but easily controllable factor in biomechanically stimulating MSC differentiation. Speculatively, other biomechanical parameters such as the stretch amplitude or the duration of stretch applied on a certain day may also have a significant impact on marker expression.

The mechanism(s) of how the two stimuli, surface matrix elasticity and applied biomechanical forces, may act together, or separately, to induce MSC differentiation is not clear. However, the elastic material properties of the extracellular matrix are proportional to the level of internal forces generated by the cytoskeleton^45^. This force balance between internal cytoskeleton contractility and external mechanical forces such as matrix elasticity or applied forces such as cyclic stretch is transduced by several mechanotransduction pathways across the mechanical continuum of ECM-integrin-cytoskeleton into the intracellular space for biological responses^45^. Thus, the mechanism of how the two stimuli, surface matrix elasticity and applied biomechanical forces, may act together is likely related to their effects on cytoskeleton contractility but further studies are necessary to examine this question in detail.

The use of growth factors and/or cytokines is a commonly used alternative method to induce myogenic differentiation of MSCs *in vitro*. Studies have used PDGF^46^,^47^,^48^,^49^ and TGF-β1 alone^46^,^47^,^48^,^50^,^51^,^52^,^53^,^54^ or in combination with L-ascorbic acid^44^,^48^,^49^,^53^,^55^. TGF-β1 has also been used in combination with biomechanical stimulation, leading to a synergistic upregulation of the smooth muscle marker gene CNN1 that was greater than the increase in response to either stimulus alone^56^. Another important study that used myogenic growth factors (PDGF and TGF-β1) together with dynamic loading demonstrated smooth muscle-specific gene and protein expression^47^. Strategies that synergistically combine different types of stimuli for inducing differentiation of MSC may prove relevant in their effectiveness. This study demonstrated that the combination of two different stimuli such as matrix stiffness and biomechanical loading was an alternative and effective method in enhancing SMC phenotype in MSCs without the use of growth factors or other soluble factors. Thus, one could conceive that combining more than two stimuli, e.g. matrix stiffness, biomechanical loading, and suitable growth factors may further enhance MSC myogenic differentiation. Such synergistic strategies may be a promising future avenue to fully utilize the MSC differentiation potential. The present study represents a significant step in this direction, and one could relatively easily incorporate our approach - combining biomechanical and biophysical stimuli - into currently available methodologies for inducing myogenic differentiation biochemically.

In conclusion, the present study demonstrated that the mechanical compression of type I collagen hydrogels generated dense viscoelastic compressed collagen sheets with a nanoscale stiffness in the range required to support myogenic differentiation. The biomechanical properties of these sheets under tensile load and the survival rate of adhering human bone marrow derived MSCs allowed the use of these sheets for cyclically stretching adhering MSCs. Using these sheets without cyclic stretch led to a pronounced increase in αSMA, transgelin, and calponin mRNA but not protein expression, compared to plastic. Cyclic tensile stimulation of MSCs adhering to the collagen type I sheets led to a significantly increased mRNA expression of αSMA, transgelin, and calponin, and also a significant increase in protein levels. This mRNA and protein expression represented an intermediate smooth muscle marker profile. Importantly, this lineage-specific differentiation was achieved without the addition of exogenous growth factors or other soluble factors. Effectively, this study established a stimulation method for inducing MSC differentiation based on combining cyclic stretch with a customized surface stiffness of compressed collagen. Techniques to obtain more targeted modulation of MSC phenotype without biochemical treatment are important in our understanding of biophysical effects on differentiation, and may have utility in developing cell-based therapies.

## Materials and Methods

All methods were carried out in accordance with the guidelines of the local research ethics committee of the medical faculty of the University of Tuebingen.

### MSC Source

Bone-marrow samples from the proximal femur were obtained with institutional approval of the local research ethics committee of the Medical Faculty of the University of Tuebingen (623/2013BO2) and with informed consent from osteoarthritic patients, who underwent total hip replacement (n = 20, age: 40 to 81 years, average 66.9 years, male = 9, female = 11) in the Department of Trauma and Restorative Surgery, BG Trauma Clinic, University of Tuebingen.

### MSC expression of cell surface antigens

The expression of CD90, CD14, CD11b (R&D Systems, Wiesbaden-Nordenstadt Germany), CD105, CD73, CD45 and CD34 (BD Pharmingen, Heidelberg, Germany) on representative MSC samples was analyzed by flow cytometry. MSCs were gently detached using Accutase (Life Technologies, Darmstadt, Germany). Unspecific binding of antibodies was blocked with Gamunex (Talecris Biotherapeutics, Frankfurt, Germany). The MSCs were washed twice with PFEA buffer (PBS, 2% FCS, 2 mM EDTA, 0.01% sodium azide) and incubated for 20 min at 4 °C with phycoerythrin (PE)-conjugated or allophycocyanin (APC)-conjugated monoclonal antibodies (mAB, BD Pharmingen). Unbound antibodies were washed away twice with PFEA buffer, and MSCs were analyzed by flow cytometry (BD LSRII, San Diego, USA). Data were evaluated using the software FlowJo (Tree Star, Inc., Ashland, Oregon, USA).

### Culture of human bone marrow MSCs

Bone marrow MSCs were prepared as described^57^. The samples were washed with PBS and centrifuged at 150 × g (10 min, room temperature). The supernatant was discarded and the pellet was re-suspended with PBS. MSCs were isolated using a Ficoll density gradient fractionation (density 1.077 g/mL, GE Healthcare Life Sciences, Uppsala, Sweden, 400 × g, 30 min, room temperature). The mononuclear cell layer was harvested, washed with PBS and seeded in T75 flasks. MSCs were expanded in good manufacturing practice (GMP)-compliant expansion medium of DMEM low glucose (Sigma-Aldrich, Hamburg, Germany) including 25 mM HEPES (Lonza Group, Basel, Switzerland), 1000 IU heparin (Carl Roth, Karlsruhe, Germany), 5% human plasma (25 ml, TCS Biosciences, Buckingham, UK), 5% human pooled platelet lysate (25 ml, 10^8^ platelets/mL medium, Blood Donation Center, University of Tuebingen, Germany), 2 mM L-glutamine (Lonza Group), and 1% penicillin-streptomycin solution (Life Technologies), as described in ref. 55. After 24 h (37 °C, 5% CO_2_), medium was discarded and replaced to remove non-attached cells and GMP expansion medium was changed every 3 to 4 days. After 5–7 days, cells were removed with trypsin, counted, and re-seeded in GMP expansion medium (passage 1, density 1.5 × 10^5^ cells/flask) for further experiments.

### MSC seeding and cell culture

After expansion in GMP expansion media as described above, MSCs were seeded at passages 2–5 at a density of 5000 MSCs/cm^2^ at day 0 onto collagen type I sheets in control medium consisting of DMEM high glucose (4 g/l, Life Technologies), 9.7% FCS, 1% penicillin-streptomycin solution, and 0.97% Amphotericin B (Biochrom, Berlin, Germany) at 37 °C and 5% CO_2_ but without pooled plasma or platelet lysate.

### Sheets of compressed collagen type I

Collagen type I sheets were produced with Amedrix (Esslingen, Germany). For collagen sheet production, collagen type I fibers were isolated from de-skinned rat tails, washed in acetic acid for 24 h, purified, and lyophilized. Rat collagen type I hydrogels with an initial collagen concentration of 8 mg/ml were generated and mechanically compressed from initial thicknesses of 10, 20, and 30 mm down to 1 mm thick sheets within a custom-made polycarbonate chamber (20.0 × 2.7 × 5.0 cm) with a porous polytetrafluoroethylene (PTFE) bottom. The PTFE pore size was 100 μm to allow water effusion during compression. Compression was achieved using various weights to generate dense compressed collagen sheets with final concentrations of 80, 160, 240 mg/ml collagen type I. In detail, 80 mg/ml sheets were generated with a weight of 2 kg applied for 2 hours, 4 kg applied overnight, 9.5 kg for 8 hours, and 27 kg overnight (total time of compression 34 hours). For 160 mg/ml sheets the final compression step applied 27 kg for 30 hours (total time of compression 52 hours). For 240 mg/ml sheets the final compression step applied 27 kg for 2.5 days (total time of compression 82 hours). The gross appearance of compressed collagen type I sheets was photographed with a digital camera and a dedicated macro lens.

### Scanning electron microscopy (SEM)

SEM was performed to characterize the collagen type I sheet surfaces. Samples were fixed overnight in 3% glutaraldehyde/PBS, additionally fixed for 60 min with 2% osmium (O_S_O_4_; Pasel und Lorei GmbH, Frankfurt, Germany), and subsequently dehydrated in ethanol. The samples were dried (critical point drying; Balzers, Critical point dryer 030, Schalksmühle, Germany), mounted, sputter-coated with gold (Ion Tech LTD, Teddington, UK), and digitally recorded in a top-down view onto the surface (Philips XL20 SEM microscope, Philips/FEI, Oregon, Hillsboro, USA).

### Atomic force microscopy (AFM)

AFM was used to characterize the nanoscale stiffness of the collagen type I sheet surfaces. The testing was performed in force spectroscopy mode by recording single force-distance-curves at the position of interest without laterally scanning the sample. Microspheres (Polybead Polystyrene 25.0 Micron Microspheres, Polysciences, Eppelheim, Germany) with 25 μm diameter were attached to a tipless cantilever (All-in-One-cantilever D, 40 N/m nominal spring constant, Budget Sensors, Sofia, Bulgaria) using the M-Bond 610-1 adhesive single kit according to the manufacturer’s instructions (Micro-Measurements, Vishay Precision Group, Wendell, USA). The cantilever of the atomic force microscope (CellHesion 200, JPK Instruments, Berlin, Germany) was calibrated on the extend curve and its spring constant was determined using the thermal noise method of the software (JPK Instruments). Samples (1 × 1 cm^2^) were measured with a maximum force of 800 nN and an extend speed of 5 μm/sec in triplicates at three locations for each sheet. The Young’s modulus was calculated using the Hertz model fit of the data processing software.

### Compressive and tensile material testing

To determine the compressive mechanical properties, five successive displacement-controlled compression ramps at 2% offset strain were applied to final strains of 3.5, 5, 6.5, 8, and 9.5% (each ramp: 60 sec compression followed by 240 sec hold) with an IncuDyn CA2008^58^,^59^. The resulting equilibrium loads at these strains were used to compute the equilibrium moduli. At 9.5% final offset strain, each sample was subjected to 3% dynamic strain amplitude at 1.0 Hz to compute the dynamic stiffness. To determine the tensile mechanical properties, each sample was subjected to 5% dynamic strain amplitude at 1.0 Hz to compute the tensile dynamic stiffness using an ElectroForce 5210 BioDynamic-Test-System (Bose, Minnesota, USA). To characterize the fatigue behavior of the collagen type I sheets, the peak load of the first and last 100 cycles of 1 hour sinusoidal stretch (5%, f = 1 Hz) was determined.

### Sinusoidal cyclic stretch

For cyclically stretching both the collagen type I sheets and the adhering MSCs, sheets (80 mg/ml) were seeded with MSCs at passages 2–5 at a density of 5000 MSCs per cm^2^ at day 0 and inserted at days 4 and 5 into the bioreactor chamber of an incubator-housed ElectroForce 5210 BioDynamic-Test-System (Bose). Samples were stretched with displacement-controlled uniaxial sinusoidal stretch (cyclic strain: 2.5 and 5%, 60 min, f = 1 Hz) for 1 h on day 4 and also on day 5 to biomechanically stimulate MSCs. The bioreactor contained 200 ml control medium (see above). Non-stimulated MSCs (adherent to non-stretched collagen type I sheets), that were seeded on day 0 and analyzed on days 4 and 5, served as controls. Thus, each individual experiment consisted of 6 collagen I sheets: 2 controls (day 4 and 5), 2 sheets (analyzed on day 4 and 5) after completion of 2.5% stretch, and 2 sheets (analyzed on day 4 and 5) after completion of 5% stretch.

### Cell Viability Staining

MSC numbers and vitality were assessed according to our published work^60^,^61^. Briefly, cells were stained with the fluorescent dyes calcein, propidium iodine, and Hoechst 33342 (Cell Viability Imaging Kit, Roche; Berlin, Germany) according to the manufacturer’s protocol. Adherent MSCs were digitally recorded in a top-down view (Zeiss LSM 510, Oberkochen, Germany; AxioVison 4.8, manual exposure correction). Using the Module MosaiX, an image mosaic consisting of 10×10 tiles (12,633 × 9,429 px corresponding to 8,211.45 × 6,128.85 μm) was reconstructed for imaging cells on cyclically stretched sheets and their controls.

### Quantitative RT-PCR

Adherent MSCs and the collagen type I sheets were digested for 4 min at 55 °C using Proteinase K (Fermentas/ThermoScientific, Waltham, MA, USA), avoiding MSC trypsinization. Total RNA was isolated using the RNA-Extraction-RNeasy-Minikit (Qiagen, Stockach, Germany). cDNA was synthesized using the Advantage RT-for-PCR Kit (Clontech, Saint-Germain-en-Laye, France). Quantitative RT-PCR was performed with the LightCycler 480 SybrGreen Master (Roche) and LightCycler 480 Probes Master (Roche) using the LightCycler 480 system and Multiwell 96Plates (Roche). Gene expression levels of α-smooth muscle actin (ACTA2), transgelin (TAGLN), calponin (CNN1), peptidylproplyl isomerase A (PPIA), and human glycerinaldehyde-3-phosphate-dehydrogenase (GAPDH) were determined. The latter two were used as reference genes. As positive controls and calibrator samples human bladder derived smooth muscle cells (HBdSMC, Promocell, Heidelberg, Germany) were used. The oligonucleotide primers used in qRT-PCR assays were 5′-TTGCCTGATGGGCAAGTGAT-3′ (forward primer sequence) and 5′-TACATAGTGGTGCCCCCTGA-3′ (reverse primer sequence) for ACTA2, 5′-AGATGGCATCATTCTTTGCGA-3′ and 5′-GCTGGTGCCAATTTTGGGTT-3′ for CNN1, 5′-CTCTGCTCCTCCTGTTCG-3′ and 5′-ACGACCAAATCCGTTGACTC-3′ for GAPDH, 5′-TTCATCTGCACTGCCAAGAC-3′ and 5′-TCGAGTTGTCCACAGTCAGC-3′ for PPIA. For TAGLN, the Qiagen assay Hs_TAGLN_2_SG was used. For TAGLN and PPIA, SybrGreen (Roche) was used. For ACTA2, CNN1, and GAPDH the Roche Universal Probe Library Probes N58 (ACTA2), N71 (CNN1), and N60 (GAPDH) were used.

### Immunofluorescence microscopy

To analyze protein expression, samples were fixed with 4% formaldehyde at 4 °C for 10 min, washed three times with PBS, and permeabilized with Triton-X100 for 20 min, washed three times with PBS, incubated for 1 h with the following primary antibodies diluted in PBS containing 0.1% BSA (Sigma-Aldrich): 1:100 mouse monoclonal anti-αSMA (Sigma-Aldrich), 1:100 rabbit polyclonal anti-transgelin (Santa Cruz Biotechnology, Heidelberg, Germany), 1:500 rabbit monoclonal anti-calponin (Abcam, Cambridge, UK). After overnight incubation at 4 °C, the samples were washed 3x with PBS, and incubated with secondary antibody (goat anti-rabbit IgG, Santa Cruz Biotechnology, dilution: 1:100) for 1 h at room temperature (except for the anti-αSMA antibody, which is labelled with Cy3). Cell nuclei were stained with DAPI (working solution: 1 μg/mL, Sigma-Aldrich), washed 3x with PBS, mounted with mounting medium (Dako Deutschland, Dako, Germany), and digitally recorded in a top-down view (Zeiss LSM 510, AxioVison 4.8, manual exposure correction).

### Collagen Type I Slot Blot and Western blot analysis

MSCs and human bladder derived smooth muscle cells (Promocell) as positive control were removed with trypsin from cell culture flasks. MSC removal from collagen sheets included Accutase treatment (Life Technologies) and manual scraping. To investigate whether collagen protein from the collagen sheets may have raised the total protein concentration in samples scratched from the collagen sheets but not in samples removed from cell culture flasks – potentially biasing the determination of protein amounts - a slot blot experiment (MilliBlot Systems, Millipore, Darmstadt, Germany) was performed for confirmation.

Cells were lysed in 200 μl protein lysis buffer (40 mM Tris/HCl pH 7.4, 300 mM NaCl, 2 mM EDTA, 2% Triton-X-100) supplemented with proteinase inhibitor at 4 °C. Insoluble material was removed by centrifugation. The protein concentration in the supernatant was determined by Bradford protein assay.

20 μg protein was added to each slot respectively and blotted onto the transfer membrane (PVDF Immobilon-P, Millipore) without denaturation in its native conformation. After 30 min incubation at room temperature the fluid was removed. After washing with Tris-Buffered Saline and Tween 20 (TBS-T) and blocking with 5% powdered milk (Carl Roth) in TBS-T, the membrane was incubated overnight at 4 °C with the primary antibody (mouse anti-collagen type I, 1:1000, Sigma-Aldrich). The membrane was washed three times with TBS-T for 15 min. Secondary antibody (horseradish peroxidase-conjugated anti-mouse pAb, 1:20,000, Jackson Immuno Research, West Grove, PA, USA) was added for 2 h at room temperature, and the membrane was washed another three times with TBS-T. Collagen type I was detected using ECL Western blotting substrate (Thermo Scientific, Waltham, MA, USA).

To determine the biomaterial- and biomechanically induced increase in SMC marker expression on protein level, Western blotting was performed. Protein samples (50 μg) were separated by 10% SDS-PAGE and transferred to a hydrophobic polyvinylidene difluoride (PVDF) membrane (Immobilon-P; Merck KGaA, Darmstadt, Germany). After blocking wih 5% powdered milk (Carl Roth, Karlsruhe, Germany) in TBS-T, membranes were incubated with primary antibodies (rabbit anti-α-smooth muscle actin (ACTA2), 1:1000 (Abcam), rabbit anti-calponin (CNN1),1:1000 (Abcam), rabbit anti-SM22 alpha (TAGLN), 1:1500 (Santa Cruz), and rabbit anti-β-tubulin, 1:2000 (Cell Signaling, Danvers, MA, USA)) with gentle shaking overnight at 4 °C according to the manufacturer’s protocols. Membranes were washed three times with TBS-T. Secondary antibody (horseradish peroxidase-conjugated anti-rabbit pAb, 1:20,000, Jackson Immuno Research) was added for 2 h at room temperature, and the membranes were washed another three times with TBS-T. Proteins were detected using ECL Western blotting substrate (Thermo Scientific) with membranes exposed to Amersham Hyperfilm ECL (GE Healthcare, Pittsburgh, PA, USA). A pre-stained protein ladder (PageRuler Plus; Thermo Scientific) was used for determination of molecular weights. For quantification ImageJ (NIH) was used.

### Statistical analyses

All data are presented as mean ± SEM except when box plots were chosen for data presentation. The box plots give the median, 25^th^ & 75^th^ as well as the 10^th^ & 90^th^ percentiles and outlying points. Calculations were performed with Microsoft Excel 2010 and SigmaPlot-11.0.0.77 (Systat, Chicago, USA). Data were analyzed for normality (Kolmogorov-Smirnov-test). Normally distributed data were subjected to the Student’s t-test; non-normal data were subjected to the Mann-Whitney-Rank-Sum-Test. More than two groups were compared using ANOVA and appropriate post-hoc tests. Differences were considered statistically significant (*) at p < 0.05.

## Additional Information

**How to cite this article**: Rothdiener, M. *et al.* Stretching human mesenchymal stromal cells on stiffness-customized collagen type I generates a smooth muscle marker profile without growth factor addition. *Sci. Rep.* **6**, 35840; doi: 10.1038/srep35840 (2016).

## Supplementary Material

Supplementary Information

## References

[b1] Bianco P. *et al.* The meaning, the sense and the significance: translating the science of mesenchymal stem cells into medicine. Nat Med 19, 35–42, doi: 10.1038/nm.3028 (2013). 23296015 PMC3998103

[b2] Caplan A. I. Adult mesenchymal stem cells for tissue engineering versus regenerative medicine. J Cell Physiol 213, 341–347, doi: 10.1002/jcp.21200 (2007). 17620285

[b3] Aicher W. K. *et al.* Regeneration of cartilage and bone by defined subsets of mesenchymal stromal cells--potential and pitfalls. Adv Drug Deliv Rev 63, 342–351, doi: 10.1016/j.addr.2010.12.004 (2011). 21184789

[b4] Sanchez-Ramos J. *et al.* Adult bone marrow stromal cells differentiate into neural cells *in vitro*. Exp Neurol 164, 247–256, doi: 10.1006/exnr.2000.7389 (2000). 10915564

[b5] Reyes M. *et al.* Origin of endothelial progenitors in human postnatal bone marrow. J Clin Invest 109, 337–346, doi: 10.1172/JCI14327 (2002). 11827993 PMC150857

[b6] Toma C., Pittenger M. F., Cahill K. S., Byrne B. J. & Kessler P. D. Human mesenchymal stem cells differentiate to a cardiomyocyte phenotype in the adult murine heart. Circulation 105, 93–98 (2002). 11772882 10.1161/hc0102.101442

[b7] Jones D. L. & Wagers A. J. No place like home: anatomy and function of the stem cell niche. Nat Rev Mol Cell Biol 9, 11–21, doi: 10.1038/nrm2319 (2008). 18097443

[b8] Morrison S. J. & Spradling A. C. Stem cells and niches: mechanisms that promote stem cell maintenance throughout life. Cell 132, 598–611, doi: 10.1016/j.cell.2008.01.038 (2008). 18295578 PMC4505728

[b9] Frenette P. S., Pinho S., Lucas D. & Scheiermann C. Mesenchymal stem cell: keystone of the hematopoietic stem cell niche and a stepping-stone for regenerative medicine. Annu Rev Immunol 31, 285–316, doi: 10.1146/annurev-immunol-032712-095919 (2013). 23298209

[b10] Bianco P. “Mesenchymal” stem cells. Annu Rev Cell Dev Biol 30, 677–704, doi: 10.1146/annurev-cellbio-100913-013132 (2014). 25150008

[b11] Park J. S. *et al.* The effect of matrix stiffness on the differentiation of mesenchymal stem cells in response to TGF-beta. Biomaterials 32, 3921–3930, doi: 10.1016/j.biomaterials.2011.02.019 (2011). 21397942 PMC3073995

[b12] Wang L. S., Chung J. E., Chan P. P. & Kurisawa M. Injectable biodegradable hydrogels with tunable mechanical properties for the stimulation of neurogenesic differentiation of human mesenchymal stem cells in 3D culture. Biomaterials 31, 1148–1157, doi: 10.1016/j.biomaterials.2009.10.042 (2010). 19892395

[b13] Engler A. J., Sen S., Sweeney H. L. & Discher D. E. Matrix elasticity directs stem cell lineage specification. Cell 126, 677–689, doi: 10.1016/j.cell.2006.06.044 (2006). 16923388

[b14] Friedl G. *et al.* Undifferentiated human mesenchymal stem cells (hMSCs) are highly sensitive to mechanical strain: transcriptionally controlled early osteo-chondrogenic response *in vitro*. Osteoarthritis Cartilage 15, 1293–1300, doi: 10.1016/j.joca.2007.04.002 (2007). 17977755

[b15] Hamilton D. W., Maul T. M. & Vorp D. A. Characterization of the response of bone marrow-derived progenitor cells to cyclic strain: implications for vascular tissue-engineering applications. Tissue Eng 10, 361–369, doi: 10.1089/107632704323061726 (2004). 15165453

[b16] Nieponice A., Maul T. M., Cumer J. M., Soletti L. & Vorp D. A. Mechanical stimulation induces morphological and phenotypic changes in bone marrow-derived progenitor cells within a three-dimensional fibrin matrix. J Biomed Mater Res A 81, 523–530, doi: 10.1002/jbm.a.31041 (2007). 17133453

[b17] Maul T. M., Chew D. W., Nieponice A. & Vorp D. A. Mechanical stimuli differentially control stem cell behavior: morphology, proliferation, and differentiation. Biomech Model Mechanobiol 10, 939–953, doi: 10.1007/s10237-010-0285-8 (2011). 21253809 PMC3208754

[b18] Yang Y., Relan N. K., Przywara D. A. & Schuger L. Embryonic mesenchymal cells share the potential for smooth muscle differentiation: myogenesis is controlled by the cell’s shape. Development 126, 3027–3033 (1999). 10357945 10.1242/dev.126.13.3027

[b19] Brown R. A., Wiseman M., Chuo C. B., Cheema U. & Nazhat S. N. Ultrarapid engineering of biomimetic materials and tissues: Fabrication of nano- and microstructures by plastic compression. Advanced Functional Materials 15, 1762–1770, doi: 10.1002/adfm.200500042 (2005).

[b20] Levis H. J. *et al.* Plastic compressed collagen as a novel carrier for expanded human corneal endothelial cells for transplantation. PLoS One 7, e50993, doi: 10.1371/journal.pone.0050993 (2012). 23226443 PMC3511456

[b21] Xiao X. *et al.* *In vivo* study of the biocompatibility of a novel compressed collagen hydrogel scaffold for artificial corneas. J Biomed Mater Res A 102, 1782–1787, doi: 10.1002/jbm.a.34848 (2014). 23813783

[b22] Braziulis E. *et al.* Modified plastic compression of collagen hydrogels provides an ideal matrix for clinically applicable skin substitutes. Tissue Eng Part C Methods 18, 464–474, doi: 10.1089/ten.TEC.2011.0561 (2012). 22195768

[b23] Bitar M., Salih V., Brown R. A. & Nazhat S. N. Effect of multiple unconfined compression on cellular dense collagen scaffolds for bone tissue engineering. J Mater Sci Mater Med 18, 237–244, doi: 10.1007/s10856-006-0685-1 (2007). 17323154

[b24] Engelhardt E. M. *et al.* Compressed collagen gel: a novel scaffold for human bladder cells. J Tissue Eng Regen Med 4, 123–130, doi: 10.1002/term.222 (2010). 19842107

[b25] Park J. S. *et al.* Differential effects of equiaxial and uniaxial strain on mesenchymal stem cells. Biotechnol Bioeng 88, 359–368, doi: 10.1002/bit.20250 (2004). 15486942

[b26] Liu B. *et al.* Role of cyclic strain frequency in regulating the alignment of vascular smooth muscle cells *in vitro*. Biophys J 94, 1497–1507, doi: 10.1529/biophysj.106.098574 (2008). 17993501 PMC2212694

[b27] Tondon A. & Kaunas R. The direction of stretch-induced cell and stress fiber orientation depends on collagen matrix stress. PLoS One 9, e89592, doi: 10.1371/journal.pone.0089592 (2014). 24586898 PMC3933569

[b28] Dominici M. *et al.* Minimal criteria for defining multipotent mesenchymal stromal cells. The International Society for Cellular Therapy position statement. Cytotherapy 8, 315–317, doi: 10.1080/14653240600855905 (2006). 16923606

[b29] Pilz G. A. *et al.* Human term placenta-derived mesenchymal stromal cells are less prone to osteogenic differentiation than bone marrow-derived mesenchymal stromal cells. Stem Cells Dev 20, 635–646, doi: 10.1089/scd.2010.0308 (2011). 21047215

[b30] Ulrich C. *et al.* Low osteogenic differentiation potential of placenta-derived mesenchymal stromal cells correlates with low expression of the transcription factors Runx2 and Twist2. Stem Cells Dev 22, 2859–2872, doi: 10.1089/scd.2012.0693 (2013). 23763516 PMC3804084

[b31] Rensen S. S., Doevendans P. A. & van Eys G. J. Regulation and characteristics of vascular smooth muscle cell phenotypic diversity. Neth Heart J 15, 100–108 (2007). 17612668 10.1007/BF03085963 PMC1847757

[b32] Beamish J. A., He P., Kottke-Marchant K. & Marchant R. E. Molecular regulation of contractile smooth muscle cell phenotype: implications for vascular tissue engineering. Tissue Eng Part B Rev 16, 467–491, doi: 10.1089/ten.TEB.2009.0630 (2010). 20334504 PMC2943591

[b33] Sobue K., Hayashi K. & Nishida W. Expressional regulation of smooth muscle cell-specific genes in association with phenotypic modulation. Mol Cell Biochem 190, 105–118 (1999). 10098977

[b34] Abreu E. L., Palmer M. P. & Murray M. M. Collagen density significantly affects the functional properties of an engineered provisional scaffold. J Biomed Mater Res A 93, 150–157, doi: 10.1002/jbm.a.32508 (2010). 19536834 PMC3069635

[b35] Hadjipanayi E., Mudera V. & Brown R. A. Guiding cell migration in 3D: a collagen matrix with graded directional stiffness. Cell Motil Cytoskeleton 66, 121–128, doi: 10.1002/cm.20331 (2009). 19170223

[b36] Cheema U. & Brown R. A. Rapid fabrication of living tissue models by collagen plastic compression: understanding three-dimensional cell matrix repair. Adv Wound Care (New Rochelle) 2, 176–184, doi: 10.1089/wound.2012.0392 (2013). 24527341 PMC3840553

[b37] Shen Z. L., Dodge M. R., Kahn H., Ballarini R. & Eppell S. J. Stress-strain experiments on individual collagen fibrils. Biophys J 95, 3956–3963, doi: 10.1529/biophysj.107.124602 (2008). 18641067 PMC2553131

[b38] Takahashi Y. & Tabata Y. Effect of the fiber diameter and porosity of non-woven PET fabrics on the osteogenic differentiation of mesenchymal stem cells. J Biomater Sci Polym Ed 15, 41–57 (2004). 15027842 10.1163/156856204322752228

[b39] Phipps M. C. *et al.* Mesenchymal stem cell responses to bone-mimetic electrospun matrices composed of polycaprolactone, collagen I and nanoparticulate hydroxyapatite. PLoS One 6, e16813, doi: 10.1371/journal.pone.0016813 (2011). 21346817 PMC3035635

[b40] Smith B. A., Tolloczko B., Martin J. G. & Grutter P. Probing the viscoelastic behavior of cultured airway smooth muscle cells with atomic force microscopy: stiffening induced by contractile agonist. Biophys J 88, 2994–3007, doi: 10.1529/biophysj.104.046649 (2005). 15665124 PMC1305393

[b41] Ito S. *et al.* Viscoelastic and dynamic nonlinear properties of airway smooth muscle tissue: roles of mechanical force and the cytoskeleton. Am J Physiol Lung Cell Mol Physiol 290, L1227–L1237, doi: 10.1152/ajplung.00299.2005 (2006). 16414980

[b42] Chaudhuri O. *et al.* Substrate stress relaxation regulates cell spreading. Nat Commun 6, 6364, doi: 10.1038/ncomms7365 (2015). PMC4518451 25695512

[b43] Ghazanfari S., Tafazzoli-Shadpour M. & Shokrgozar M. A. Effects of cyclic stretch on proliferation of mesenchymal stem cells and their differentiation to smooth muscle cells. Biochem Biophys Res Commun 388, 601–605, doi: 10.1016/j.bbrc.2009.08.072 (2009). 19695226

[b44] Brun J. *et al.* Smooth muscle-like cells generated from human mesenchymal stromal cells display marker gene expression and electrophysiological competence comparable to bladder smooth muscle cells. PLoS One 10, e0145153, doi: 10.1371/journal.pone.0145153 (2015). 26673782 PMC4684225

[b45] Sun Y., Chen C. S. & Fu J. Forcing stem cells to behave: a biophysical perspective of the cellular microenvironment. Annu Rev Biophys 41, 519–542, doi: 10.1146/annurev-biophys-042910-155306 (2012). 22404680 PMC4123632

[b46] Bharadwaj S. *et al.* Characterization of urine-derived stem cells obtained from upper urinary tract for use in cell-based urological tissue engineering. Tissue Eng Part A 17, 2123–2132, doi: 10.1089/ten.TEA.2010.0637 (2011). 21513463 PMC9836685

[b47] Tian H. *et al.* Myogenic differentiation of human bone marrow mesenchymal stem cells on a 3D nano fibrous scaffold for bladder tissue engineering. Biomaterials 31, 870–877, doi: 10.1016/j.biomaterials.2009.10.001 (2010). 19853294 PMC2787773

[b48] Narita Y., Yamawaki A., Kagami H., Ueda M. & Ueda Y. Effects of transforming growth factor-beta 1 and ascorbic acid on differentiation of human bone-marrow-derived mesenchymal stem cells into smooth muscle cell lineage. Cell Tissue Res 333, 449–459, doi: 10.1007/s00441-008-0654-0 (2008). 18607632

[b49] Antoon R., Yeger H., Loai Y., Islam S. & Farhat W. A. Impact of bladder-derived acellular matrix, growth factors, and extracellular matrix constituents on the survival and multipotency of marrow-derived mesenchymal stem cells. J Biomed Mater Res A 100, 72–83, doi: 10.1002/jbm.a.33230 (2012). 21972045

[b50] Gong Z., Calkins G., Cheng E. C., Krause D. & Niklason L. E. Influence of culture medium on smooth muscle cell differentiation from human bone marrow-derived mesenchymal stem cells. Tissue Eng Part A 15, 319–330, doi: 10.1089/ten.tea.2008.0161 (2009). 19115826 PMC2716410

[b51] Bajpai V. K., Mistriotis P. & Andreadis S. T. Clonal multipotency and effect of long-term *in vitro* expansion on differentiation potential of human hair follicle derived mesenchymal stem cells. Stem Cell Res 8, 74–84, doi: 10.1016/j.scr.2011.07.003 (2012). 22099022 PMC3222855

[b52] Harris L. J. *et al.* Differentiation of adult stem cells into smooth muscle for vascular tissue engineering. J Surg Res 168, 306–314, doi: 10.1016/j.jss.2009.08.001 (2011). 19959190 PMC2888621

[b53] Williams C. *et al.* A comparison of human smooth muscle and mesenchymal stem cells as potential cell sources for tissue-engineered vascular patches. Tissue Eng Part A 18, 986–998, doi: 10.1089/ten.TEA.2011.0172 (2012). 22145703

[b54] Kurpinski K. *et al.* Transforming growth factor-beta and notch signaling mediate stem cell differentiation into smooth muscle cells. Stem Cells 28, 734–742, doi: 10.1002/stem.319 (2010). 20146266

[b55] Brun J., Abruzzese T., Rolauffs B., Aicher W. K. & Hart M. L. Choice of xenogenic-free expansion media significantly influences the myogenic differentiation potential of human bone marrow-derived mesenchymal stromal cells. Cytotherapy 18, 344–359, doi: 10.1016/j.jcyt.2015.11.019 (2016). 26857228

[b56] Kurpinski K., Chu J., Wang D. & Li S. Proteomic profiling of mesenchymal stem cell responses to mechanical strain and TGF-beta1. Cell Mol Bioeng 2, 606–614, doi: 10.1007/s12195-009-0090-6 (2009). 20037637 PMC2792360

[b57] Felka T., Schafer R., Schewe B., Benz K. & Aicher W. K. Hypoxia reduces the inhibitory effect of IL-1beta on chondrogenic differentiation of FCS-free expanded MSC. Osteoarthritis Cartilage 17, 1368–1376, doi: 10.1016/j.joca.2009.04.023 (2009). 19463979

[b58] Rolauffs B. *et al.* Stress-vs-time signals allow the prediction of structurally catastrophic events during fracturing of immature cartilage and predetermine the biomechanical, biochemical, and structural impairment. J Struct Biol 183, 501–511, doi: 10.1016/j.jsb.2013.06.011 (2013). 23810923 PMC3816199

[b59] Rolauffs B. *et al.* Vulnerability of the superficial zone of immature articular cartilage to compressive injury. Arthritis Rheum 62, 3016–3027, doi: 10.1002/art.27610 (2010). 20556809 PMC2952071

[b60] Rolauffs B. *et al.* Onset of preclinical osteoarthritis: the angular spatial organization permits early diagnosis. Arthritis Rheum 63, 1637–1647, doi: 10.1002/art.30217 (2011). 21630246

[b61] Rolauffs B. *et al.* Proliferative remodeling of the spatial organization of human superficial chondrocytes distant from focal early osteoarthritis. Arthritis Rheum 62, 489–498, doi: 10.1002/art.27217 (2010). 20112377 PMC2945301

