# Supplementary material for: Stretching human mesenchymal stromal cells on stiffness-customized collagen type I generates a smooth muscle marker profile without growth factor addition
Source: Sci Rep. 2016 Oct 24;6:35840. doi: 10.1038/srep35840 (PMC5075785; doi:10.1038/srep35840)

# Stretching human mesenchymal stromal cells on stiffness-customized collagen type I generates a smooth muscle marker profile without growth factor addition

[1,$] Miriam Rothdiener, [2,$] Miriam Hegemann, [1] Tatiana Uynuk-Ool , [3] Brandan Walters, [1] Piruntha Papugy, [1] Phong Nguyen, [1] Valentin Claus, [4] Tanja Seeger, [5] Ulrich Stoeckle, [6] Karen A. Boehme, [2] Wilhelm K. Aicher, [3] Jan P. Stegemann, [8] Melanie L. Hart, [7] Bodo Kurz, [4] Gerd Klein, [8] Bernd Rolauffs*

Supplementary Information

The Western blots depicted in Figure 7C of the main manuscript have been cropped from two gels. The depicted lanes for β-tubulin, calponin, and transgelin were cropped from a single Western blot labeled A, and the lanes depicted for αSMA and β-tubulin were cropped from another Western blot before (αSMA) and after stripping (β-tubulin) labeled B. Lanes depicted in the main manuscript are marked here by a black font, lanes not depicted in the main manuscript are marked in light grey. The molecular weight markers were manually marked by black dots.

I: MSCs on Cell Culture Flasks
II: SMCs on Cell Culture Flasks
III: MSCs on Collagen I Sheets (no stretch)
IV: MSCs on Collagen I Sheets (5 % stretch, day 5)

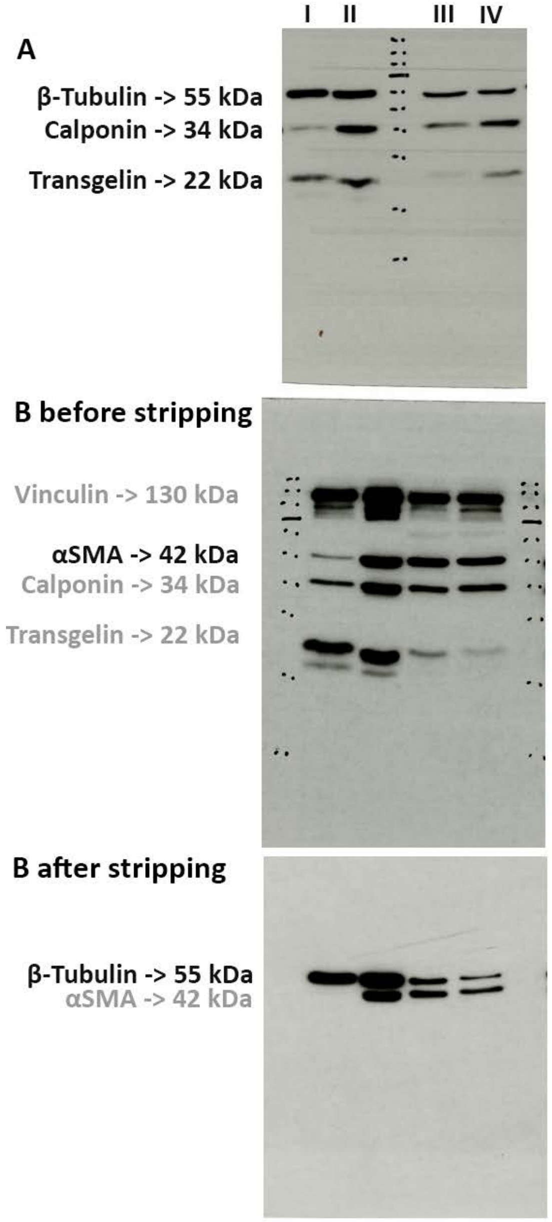

Supplement: Supplementary Information [file srep35840-s1.pdf]
